# Adult Spinal Cord Injury without Radiographic Abnormalities (SCIWORA): Clinical and Radiological Correlations

**DOI:** 10.4021/jocmr2009.08.1256

**Published:** 2009-08-20

**Authors:** Siddhartha Sharma, Manjeet Singh, Iftikhar H Wani, Sushil Sharma, Narendra Sharma, Dara Singh

**Affiliations:** a Postgraduate Department of Orthopedics, Government Medical College, Jammu, India; b Postgraduate Department of Radiodiagnosis, Government Medical College, Jammu, India

## Abstract

**Background:**

This study is aimed to determine the clinical and radiological corellations of adult patients with Spinal Cord Injury Without Radiographic Abnormalities (SCIWORA).

**Methods:**

The study population consisted of all adult patients with suspected cervical spine injury. SCIWORA was defined as the presence of either no injury or a neural injury on Magnetic Resonance Imaging (MRI) in the absence of radiographic or Computed Tomographic (CT) Scan findings suggestive of trauma in patients with neurological deficit. Purely extra neural compressive lesions were excluded from the study.

**Results:**

Twelve of ninety seven (12.4%) patients had a neural injury on MRI with normal radiographs and CT scan. These included cord contusion in five cases, cord edema in five cases and cord hemorrhage in two cases. Ten patients were managed conservatively and two patients with disc prolapse were managed surgically. All patients showed at least one ASIA Impairment Scale (AIS) grade improvement and three patients (25%) recovered completely.

**Conclusions:**

Parenchymal spinal cord injury is the single most important determinant in the long term outcome of adult SCIWORA patients. Cord hemorrhage has the worst prognosis and cord edema has the best. Longitudinal signal extension and associated extra neural injuries are also associated with poorer outcomes. Cases with purely neural injuries can be managed conservatively, but associated extra neural injuries, especially disc prolapse and ligamentous instability, warrant surgical management.

**Keywords:**

Post Traumatic Myelopathy; Spinal Cord Trauma; Computed tomography; Magnetic resonance imaging; SCIWORA

## Introduction

Spinal cord injury without radiographic abnormalities (SCIWORA) has been classically described in pediatric cervical spine injuries. This acronym was coined by Pang and Wilberger in 1982 to denote presence of clinical findings (neurological deficit) in the setting of normal X Rays and Computerized Tomographic (CT) Scan. The advent of Magnetic Resonance Imaging (MRI) has made it possible to accurately characterize the underlying spinal cord injury even when radiographs and CT scan are normal. In view of these advances, Pang reviewed his work in 2004 and advocated that only cases with neural injuries seen on MRI or those with normal MRI findings should be counted as SCIWORA and purely extra neural compressive lesions should be excluded from the definition of SCIWORA.

Adult SCIWORA is a rare phenomenon and not much is known about its exact pathophysiology. This study was done with the aims to describe the clinical and MRI findings of adult SCIWORA and to discuss the management of this unique condition.

## Methods

The prospective study was conducted in our institution, a tertiary referral centre for spine injuries from January 2005 to July 2008. All adult (> 20 years of age) cervical spine injury patients presenting to our institution were provisionally included and then evaluated for SCIWORA.

On admission, all patients were managed as per Advanced Trauma Life Support (ATLS) protocols. Immediate immobilization, if not done already, was achieved using a Philadelphia collar. This was followed by a thorough neurological examination. Neurodeficit was graded using ASIA Impairment Scale (AIS).

All patients were screened by AP, lateral and mouth open radiographs of the cervical spine. CT scanning was done using 2-slice Spiral CT in cases where radiographs were equivocal and in cases of suspected injuries to upper cervical spine (C1-2) and cervicothoracic junction. MRI was done in all cases with neurodeficit using T1 weighted (T1W) saggital, T2 weighted (T2W) saggital and axial views.

**Figure 1:**
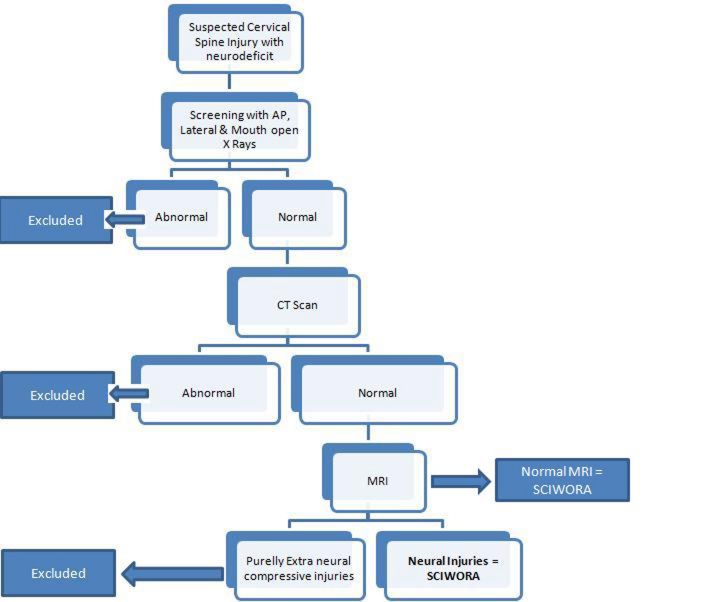
Algorithm for diagnosis of adult SCIWORA.

Using the criteria described by Pang et al, SCIWORA was defined as presence of either no lesion or a neural lesion on MRI in the setting of normal radiographs and CT scan in patients with neurological deficit [1]. Purely extra neural compressive lesions with no intra neural pathology were excluded from the study (Fig. 1).

We used the classification described by Kulkarni et al to classify the neural injuries [2]. Pattern I (Hemorrhage) is defined as a large central area of hypointensity surrounded by a thin rim of hyperintensity on T2W images. Pattern II (Edema) is characterized by an area of hyperintensity on T2W images. Pattern III (Cord contusion) is characterized by a thin area of central hypointensity and thick rim of hyperintensity on T2W images.

For the purpose of the study, the following radiological parameters were defined as Abnormal and such patients were excluded from the study: (1) frank fractures, dislocations and subluxations; (2) alignment of vertebral bodies: was assessed using anterior and posterior cortical lines of the vertebral body and the spinolaminar line; (3) the laminar space: to detect rotational injuries; (4) prevertebral soft tissue thickness: thickness > 5 mm opposite C3 and > 20mm opposite C6 was taken as abnormal; (5) atlantooccipital alignment: was assessed using the Basion dental interval (> 12 mm considered abnormal) and Basionaxial interval (> 12 mm considered abnormal); (6) atlantoaxial alignment: was assessed using the Anterior Atlanto Dens Interval (AADI), and AADI of > 3 mm was taken as abnormal; (7) Spinal Canal diameter: was assessed using the AP width of spinal canal : vertebral body ratio, and ratios < 0.8 were taken as abnormal.

Presence of degenerative / spondylotic changes was considered as normal. Methylprednisolone therapy was instituted, wherever applicable as per North American Spinal Cord Injury Study III (NASCIS III) criteria. Immobilization was continued in a Philadelphia collar for a total duration of 6 weeks. The patients and their attendants were educated about log rolling precautions, prevention of bedsores and care of bowel and bladder. Physiotherapy was instituted as soon as the general condition of the patient permitted and continued throughout the course of treatment.

Surgical decompression, in the form of anterior cervical discectomy and fusion, was reserved for cases of intervertebral disc prolapse. Follow-up was done monthly till 6 months, every two months for the next 6 months and every six months thereafter. Flexionextension views were obtained in all cases at 12 weeks to check for instability. The patients were assessed using AIS for neurological recovery and presence of complications.

## Results

There were a total of 97 adult patients with suspected cervical spine injury and neurological deficit. Radiographs and CT scan were normal in 15 patients. One patient died before MRI studies could be performed and she was excluded from the study. This was a seventy years old female who had associated subarachnoid hemorrhage and left sided haemothorax.

Two patients with normal radiographs and CT scan revealed intervertebral disc prolapse with cord compression and no intra neural pathology. Both were excluded from the study.

**Figure 2:**
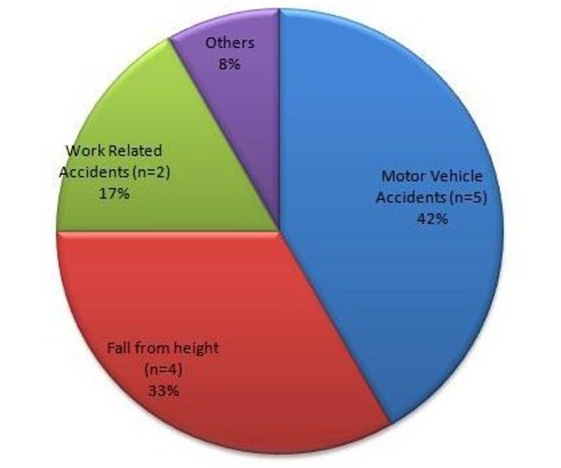
Pie diagram showing etiology of adult SCIWORA.

Using our operational definition of SCIWORA, we identified twelve cases. Of these, ten were males and two were females. The most common mode of injury was motor vehicle accident (n = 5) followed by fall from height (n = 4). The other cases were due to work related accidents (n = 2) and roof collapse (n = 1) (Fig. 2). Six of the twelve patients were smokers, four had a history of hypertension whereas one had Type 2 Diabetes Mellitus and was on oral hypoglycaemic medication.

**Figure 3:**
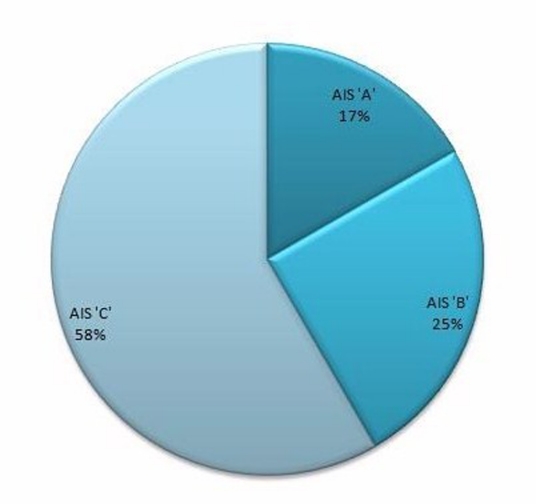
Pie diagram showing neurological deficit at presentation graded by ASIA Injury Score (AIS).

Complete spinal cord injury was present in two cases. Of the incomplete spinal cord injury patients, seven were AIS C and three were AIS B. Central cord syndrome was present in one case (Fig. 3).

**Figure 4:**
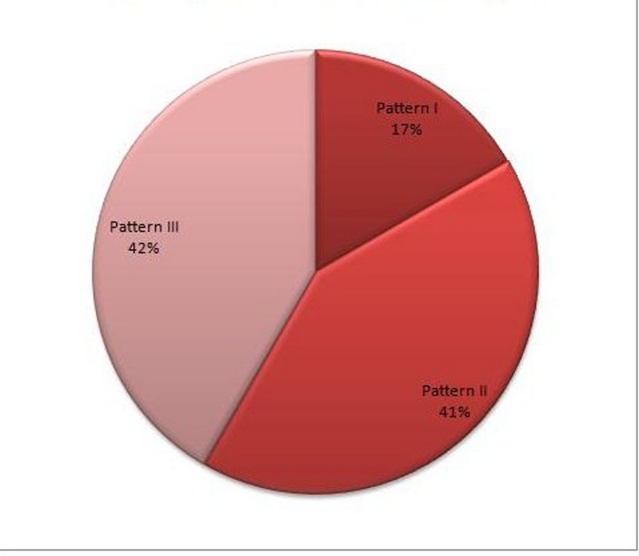
Pie diagram showing the distribution of parenchymal spinal cord injury.

Radiographs showed varying degrees of spondylosis in 41.7% of the cases (n = 5). The neural injuries were cord contusion (Pattern III) in 41.5% (n = 5), cord edema (Pattern II) in 41.5 % cases (n = 5) and cord hemorrhage (Pattern I) in the remaining 17% cases (n = 2) (Fig. 4). The extra neural injuries included ligamentum flavum bulging in four cases, multiple level disc intervertebral disc bulges / prolapse in four cases and single level disc prolapse in one case (Table 1).

**Table 1:** Patients data

Sno.	Age/Sex	Mode of Trauma	Neurological Status at Admission (AIS Grade)	Radiographs & CT Scan	Neural Injury (On MRI)	Extra Neural Injury (On MRI)	Treatment	Neurological Status at 12 months (AIS Grade)
1.	22 M	MVA	AIS ‘A’	Normal	Cord contusion opposite C6,7 Pattern III	Disc bulges at C 3-4 & C6-7, L. Flavum bulging C6-7.	Conservative	AIS ‘C’
2.	32 M	Roof Collapse	AIS ‘C’	Normal	Cord contusion with long segment cord edema C2-7 Pattern III	L. Flavum bulge C4-5.	Conservative	AIS ‘D’
3.	55 M	MVA	AIS ‘B’	Spondylosis	Cord Hemorrhage. C5-6 Pattern I	Disc Prolapse C5-6 & C6-7. L.Flavum bulging from C 5-7.	Discectomy C5-6, C6-7 and Fusion. (Tricortical Iliac Graft & 2 level CSLP)	AIS ‘C’
4.	51 M	MVA	AIS ‘C’ Central Cord Syndrome	Spondylosis	Cord edema C1-2. Pattern II	Multiple level disc bulges, L. Flavum bulge C4-5.	Conservative	AIS ‘D’
5.	58 M	Fall from height	AIS ‘C’	Spondylosis	Cord edema C4-6 Pattern II	-	Conservative	AIS ‘E’
6.	33 F	Fall from Height	AIS ‘C’	Normal	Cord edema C5-6 Pattern II	-	Conservative	AIS ‘E’
7.	29 M	WRA	AIS ‘B’	Spondylosis	Cord Contusion C6-7 Pattern III	Multiple Level Disc Bulges	Conservative	AIS’D’
8.	40 M	Fall from height	AIS ‘C’ Central Cord Syndrome	Normal	Cord Contusion C4-6 Pattern III	-	Conservative	AIS ‘D’
9.	52 F	MVA	AIS ‘C’	Spondylosis	Cord edema C5-6 Pattern II	Disc Prolapse C5-6.	Discectomy C5-6 & Fusion (Tricortical iliac graft &1 level CSLP)	AIS ‘D’
10.	38 M	MVA	AIS ‘A’	Normal	Cord Hemorrhage C4-6 Pattern I	-	Conservative	AIS ‘B’
11.	26 M	WRA	AIS ‘B’	Normal	Cord Contusion C4-5 Pattern III	-	Conservative	AIS ‘D’
12.	28 M	Fall from Height	AIS ‘C’	Normal	Cord edema C4-6 Pattern II	-	Conservative	AIS ‘E’

MVA: Motor vehicle accident; WRA: Work related accident; CSLP: Cervical Spine Locking Plate.

**Figure 5:**
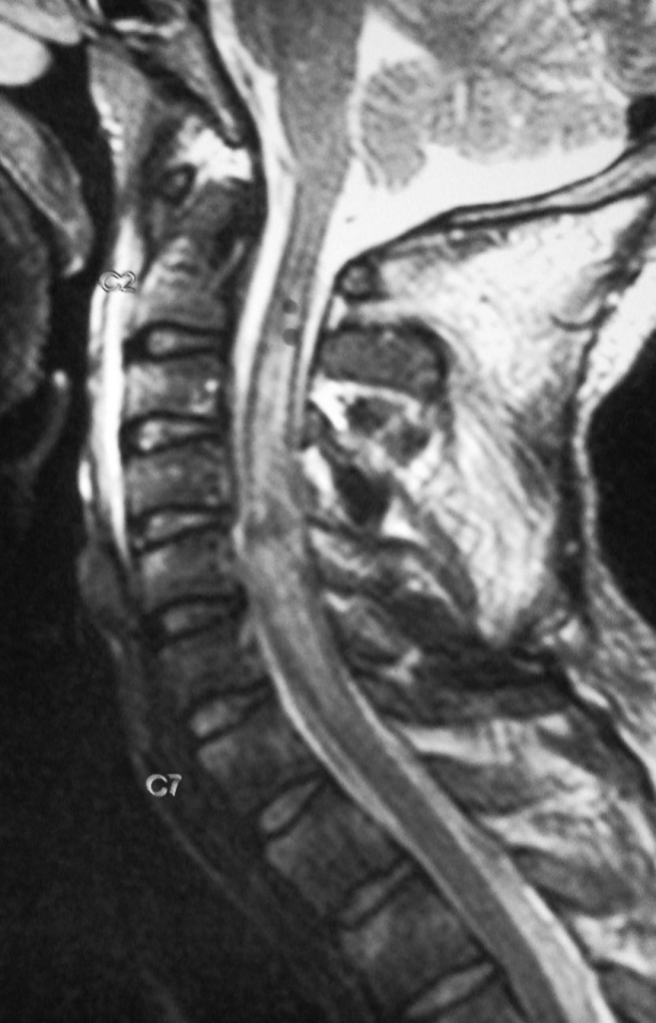
T2W saggital MR image of a 32 year old male showing long segment cord edema from C2-7 levels.

Longitudinal extension of the abnormal signal was noted in one case. This was a 32 years old male who was injured in roof collapse. MRI revealed cord contusion and a long segment cord edema (Pattern III) from C2-7. He developed respiratory distress on the second day of admission. He required ventilatory support for a week but subsequently recovered (Fig. 5).

Methylprednisolone as per NASCIS III criteria was administered to seven of the twelve cases. Anterior cervical discectomy and fusion with tricortical iliac graft and cervical spine locking plate were done in two cases with traumatic disc prolapse.

Both patients with anterior discectomy and fusion had an uneventful post operative course. Good fusion was achieved and there were no instances of hardware failure. Flexionextension views taken at 12 weeks post-injury were normal in all cases.

**Figure 6:**
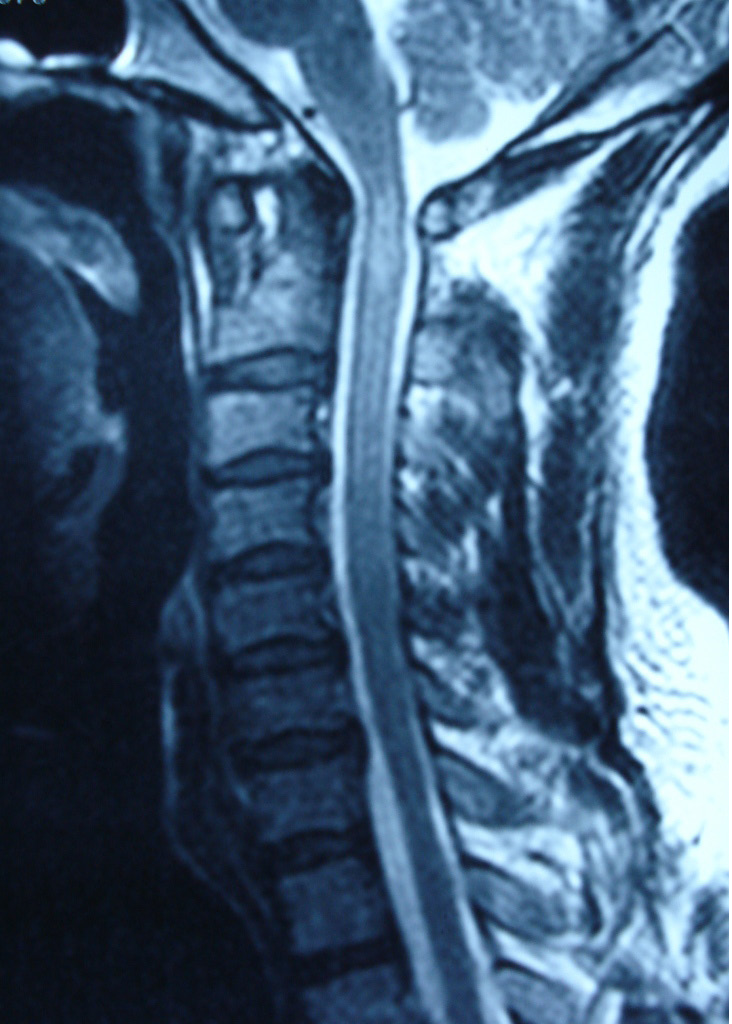
T2W saggital MR image of a 51 year old male showing cord edema opposite C1-2 levels. This patient recovered completely.

At the end of one year follow-up, all patients showed at least one AIS grade improvement and three patients (25%) recovered completely. All of these three cases had cord edema. Only one patient with cord edema did not show full recovery. This was a 52 years old male with cord edema opposite C1-2 and multiple level disc bulges. He improved from AIS C to AIS D and continues to have weakness in the left ankle and foot muscles (Fig. 6).

**Figure 7:**
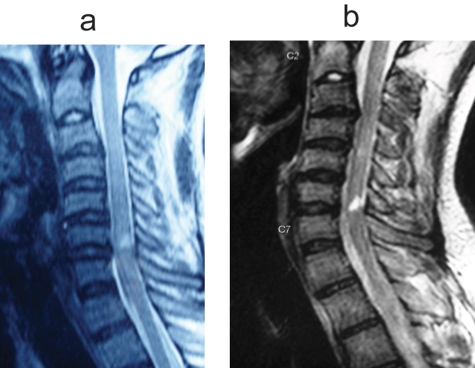
(a) T2W saggital MR image of a 22 year old male showing cord contusion at C6-7 level and disc bulging at C3-4 and C6-7 levels. (b) T2W saggital MR image of the same patient done after one year. Note the focal myelomalacia at C6-7 level.

**Figure 8:**
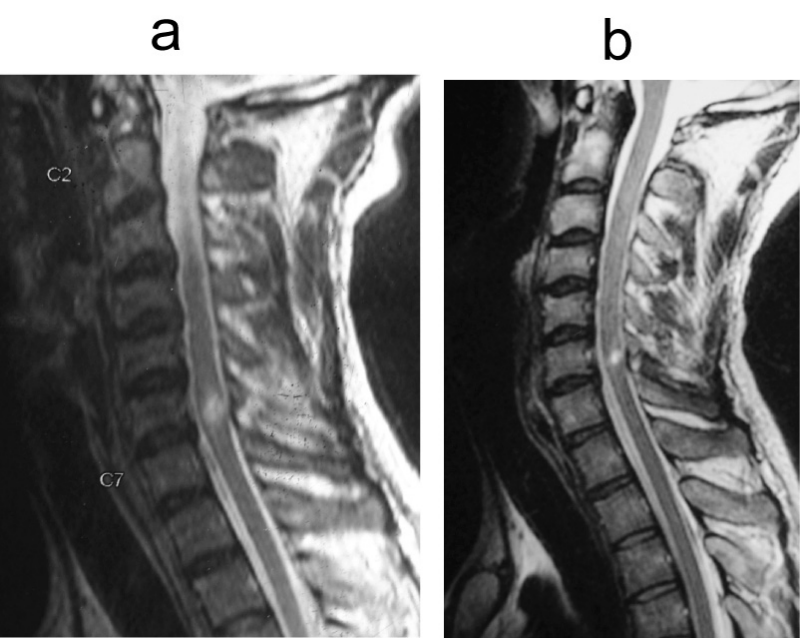
(a) T2W saggital MR image of a 29 year old male showing cord contusion opposite C6-7 level and multiple disc bulges. (b) T2W saggital MR image of the same patient done after one year. The contusion has decreased in size and there is no myelomalacia.

Follow-up MRI after one year of injury were done in two cases of cord contusion. One was a 29 years old male with cord contusion opposite C6-7 levels and multiple level disc bulges (Fig. 7a). At one year of follow-up, repeat MRI showed that the contusion had significantly decreased in size and there was no myelomalacia (Fig. 7b). This patient improved from AIS B to AIS D. The other case was that of a 22 years old with cord contusion opposite C6-7 and disc bulges at two levels (Fig. 8a). Repeat MRI after one year showed focal myelomalacia at C6-7 level (Fig. 8b). He had complete cord injury at presentation (AIS A) and improved only to AIS C. The average duration of follow-up was 2.0 years (1.2 - 3 years).

## Discussion

SCIWORA was described by Pang and Wilberger in 1982 in the pediatric cervical spine. Inherent elasticity of the vertebral column due to ligamentous laxity, combined with the large head size predisposes the pediatric cervical spine to deforming forces. Pang described four mechanisms of SCIWORA viz. flexion, hyperextension, longitudinal distraction and ischemia [3].

Tator has recommended that the term spinal cord injury without radiologic evidence of trauma (SCIWORET) has been used for the adult population since many cases have preexisting spondylotic changes [4]. With advances in imaging and the use of multi detector CT scan, the incidence of SCIWORET in adults has decreased from 14% to 5% [5, 6].

Though the most common site of involvement is cervical spine, the thoracic spinal cord may also be involved. Launay et al in a meta analysis of 353 pediatric SCIWORA cases found that thoracic spinal cord was involved in 26% of the cases [7].

SCIWORA in adults is a rare phenomenon. A review of Medline revealed less than 100 cases. In a series of 40 adult patients, Tewari et al reported 12% incidence of SCIWORA [8]. The incidence in our series was similar (12.4%).

The pathophysiology of adult SCIWORA is quite different from that of pediatric population. Most cases are due to hyperextension injury to the spine, which may be seen in rear end motor vehicle collisions or direct anterior craniofacial trauma. Another peculiar feature is preexisting cervical spondylosis, which predisposes to cord injury even with trivial trauma. Kasimatis et al have suggested that elderly patients with spondylosis have posterior vertebral spurs and ligamentum flavum bulging due to decreased vertebral body height. Hyperextension injury in such cases, even if trivial, can result in a central cord syndrome [9]. In our series, varying degrees of cervical spondylosis were observed in 80% of the cases and all cases demonstrated bulging of the ligamentum flavum.

Schneider et al described the central cord syndrome in patients with narrow cervical canals who sustained a hyperextension injury. The cervical spinal cord gets compressed between the posterior vertebral body spurs and the buckled ligamentum flavum. The typical presentation is that of neurodeficit which is more severe in upper limbs as compared to the lower limbs [10]. Of all the incomplete cord syndromes, the central cord syndrome has the worst prognosis and Brown Sequard syndrome has the best [11].

The various criteria for radiographic evaluation of the cervical spine were described by Weir [12]. One of these criteria is the prevertebral soft-tissue shadow, which should not exceed 5 mm in width at the level of the anteroinferior border of the third cervical vertebra. A width of more than 5 mm strongly suggests injury with soft-tissue swelling.

However, in a study of 212 patients, Herr et al. showed that prevertebral soft-tissue measurement at C3 is an insensitive marker of cervical spine fracture or dislocation and does not correlate with the location or mechanism of injury. Loss of the cervical lordotic curve is not in its own evidence of cervical spine injury with resultant muscle spasm, it may be simply a normal variant [13].

Lateral flexion and extension views can be made to determine the stability of the cervical spine, but these are not routinely recommended in the initial examination. Ralston et al. noted that flexion and extension views were unlikely to show acute instability initially [14]. Pollack et al. recommended of waiting 2 to 4 weeks before obtaining flexion and extension radiographs to avoid the problems of muscle spasm [15]. In our series, flexionextension radiographs were done only after 12 weeks of injury.

Ruling out cervical spine injury can be extremely difficult in obtunded patients. We recommend that cervical spine injury should be presumed as present in all patients with head injury unless proved otherwise and immobilization done even if radiographs are normal. Griffen et al. reviewed more than 1000 patients with blunt trauma and average Glasgow Coma Scale score of 13 and Injury Severity Score of 8.5. They concluded that cervical CT should replace conventional radiography in the evaluation of the cervical spine in patients with blunt trauma [16].

However, in a developing country like ours, the importance of conventional radiographs cannot be under estimated. We used CT scan only in patients with equivocal radiographic findings and in case of suspected injuries of upper cervical spine and cervico-dorsal junction.

MRI has helped identify the subtle spinal cord parenchymal abnormalities and ligamentous disruptions. Liao et al described four patterns of parenchymal cord injuries viz. concussion, edema, contusion and transection [17].

Cord concussion is characterized by biochemical changes within the spinal cord and is associated with normal MRI signals. It probably represents SCIWORA in the strictest sense. We did not come across any patients with normal MRI findings. Cord hemorrhage appears as an area of central large hypointensity surrounded by thin rim on hyperintensity T2W sequences. Cord edema is identified by hyperintense signal on T2W sequences. Cord concussion appears as an area of small central hypointensity surrounded by larger peripheral rim of hyperintensity. The various extra parenchymal injuries include annular tears with resultant disc bulge or prolapse, rupture of the anterior longitudinal ligament and bulging of the ligamentum flavum [18].

Shen et al have described the use of diffusion weighted MRI (DWI) in the diagnosis of spinal cord injury [19]. Patients with normal signals on MRI show hyperintense signals on DWI. Thus, it may be a useful tool in cases of cord concussions. Pang et al have described the use of Somato Sensory Evoked Potentials (SSEP) for the diagnosis of SCIWORA [1]. Kamoto et al have evaluated the use of fluorodeoxyglucose (FDG)-positron emission tomography (PET) imaging and suggested that FDG-PET imaging can be used to visualize deterioration of cervical spinal cord function [20]. In spite of all these advances, MRI remains the most commonly used diagnostic tool in day to day practice.

From our study, cord edema seemed to have the best prognosis. However, one patient had extension of abnormal signal from C2-7, which was associated with a poorer outcome. Associated extra parenchymatous injuries have poorer outcomes and cord hemorrhage has the worst outcome. This is consistent with the works of Kulkarni, Pang, Tewari and Kasimatis [1, 2, 8, 9].

Methylprednisolone acts by obtunding the secondary injury cascade. We followed NASCIS III protocols for our study but the number of patients is too small to comment on the efficacy. However, there were no steroid induced complications in our study.

Kasimatis et al in their retrospective series of 6 patients with adult SCIWORA performed surgical decompression and stabilization in all cases. In our study, purely neural injuries and mixed neural and extra neural injuries with no cord compression were managed conservatively. We achieved good to excellent results, except in cases with cord hemorrhage. We reserved surgical treatment only for mixed neural and extra neural injuries with disc cord compression.

The present study has its limitations. Though it is a prospective study, the number of patients was small. This is attributable to the rare nature of this condition. However, ours is the second largest prospective study conducted so far and we have the longest duration of follow-up of Adult SCIWORA patients. The study also highlights the need for international collaboration in order to conduct a large, multicenter prospective study comparing the various modalities of treatment, keeping in view its rare nature.

In conclusion, adult SCIWORA comprises of a three patterns of neural injuries viz. hemorrhage, edema and contusion. Cord edema has the best prognosis and cord haemorrhage has the worst. Longitudinal signal extension and multiple extra neural injuries are associated with poorer outcomes. Purely extra neural compressive pathologies are best regarded as separate entities and should not be merged with SCIWORA. Conservative management of purely neural injuries yields good results, but the presence of coexistent extra neural injuries, especially disc prolapse and ligamentous instability requires surgical management.

## Abbreviations

AIS: ASIA Impairment Scale
ASIA: American Spinal Injuries Association
ATLS: Advanced Trauma Life Support
CSLP: Cervical Spine Locking Plate
CT: Computerized Tomography
DWI: Diffusion Weighted MRI
FDG PET: Fluorodeoxy Glucose Positron Emission Tomography
MRI: Magnetic Resonance Imaging
MVA: Motor Vehicle Accident
NASCIS III: North American Spinal Cord Injury Study III
SCIWORA: Spinal Cord Injury Without Radiographic Abnormalities
SCIWORET: Spinal Cord Injury Without Radiologic Evidence of Trauma
SSEP: Somato Sensory Evoked Potentials
T1W Sag: T1 weighted saggital image
T2W Sag: T2 weighted saggital image
